# Feral swine as sources of fecal contamination in recreational waters

**DOI:** 10.1038/s41598-021-83798-6

**Published:** 2021-02-18

**Authors:** Anna M. McKee, Paul M. Bradley, David Shelley, Shea McCarthy, Marirosa Molina

**Affiliations:** 1 South Atlantic Water Science Center, U.S. Geological Survey, 1770 Corporate Drive Suite 500, Norcross, GA 30093 USA; 2 South Atlantic Water Science Center, U.S. Geological Survey, 720 Gracern Rd., Suite 129, Columbia, SC 29210 USA; 3 National Park Service, Congaree National Park, 100 National Park Rd, Hopkins, SC 29061 USA; 4 Department of Environmental Health Sciences, University of South Carolina, 921 Assembly St, Columbia, SC 29201 USA; 5 Office of Research and Development, U.S. Environmental Protection Agency, 109 T.W. Alexander Dr, Research Triangle Park, NC 27709 USA

**Keywords:** Applied microbiology, Invasive species

## Abstract

Recreational waters are primary attractions at many national and state parks where feral swine populations are established, and thus are possible hotspots for visitor exposure to feral swine contaminants. Microbial source tracking (MST) was used to determine spatial and temporal patterns of fecal contamination in Congaree National Park (CONG) in South Carolina, U.S.A., which has an established population of feral swine and is a popular destination for water-based recreation. Water samples were collected between December 2017 and June 2019 from 18 surface water sites distributed throughout CONG. Host specific MST markers included human (HF183), swine (Pig2Bac), ruminant (Rum2Bac), cow (CowM3), chicken (CL), and a marker for shiga toxin producing *Escherichia coli* (STEC; *stx2*). Water samples were also screened for culturable *Escherichia coli* (*E. coli*) as part of a citizen science program. Neither the cow nor chicken MST markers were detected during the study. The human marker was predominantly detected at boundary sites or could be attributed to upstream sources. However, several detections within CONG without concurrent detections at upstream external sites suggested occasional internal contamination from humans. The swine marker was the most frequently detected of all MST markers, and was present at sites located both internal and external to the Park. Swine MST marker concentrations ≥ 43 gene copies/mL were associated with culturable *E. coli* concentrations greater than the U.S. Environmental Protection Agency beach action value for recreational waters. None of the MST markers showed a strong association with detection of the pathogenic marker (*stx2*). Limited information about the health risk from exposure to fecal contamination from non-human sources hampers interpretation of the human health implications.

## Introduction

Feral swine fecal contamination in environmental waters and wastewater is recognized as a possible exposure route for disease transmission^1^ from zoonotic pathogens such as Hepatitis E virus, *Cryptosporidium parvum*, and *Giardia*^2–4^. While reports to date from the U.S. and other countries do not show patterns of significant disease outbreaks specifically linked to feral swine impacts on water quality, this may provide a false perception of risk. Most of the feral swine range expansion and population increases in the U.S. have largely occurred within the last 20 years^5^.

Feral swine have become abundant and widespread in the United States (U.S.) because of their ability to adapt to a wide range of habitats and their high reproductive potential. Commonly reported environmental impacts from feral swine include physical habitat alterations resulting from rooting behavior (i.e., disturbing surfaces with their snouts to move objects around), predation of native species, and resource competition with native species^5–7^. Despite feral swine often inhabiting and defecating in areas close to water^5,8,9^, the impacts of swine fecal contamination in recreational waters and potential human health risks are still poorly understood^9–12^.

To prevent illnesses from exposure to pathogens from fecal contamination in waters designated for drinking and recreation, state governments set regulatory limits for fecal indicator bacteria (FIB) based on U.S. Environmental Protection Agency (EPA) recommendations. When regulatory limits are exceeded, the water body is listed as impaired and authorities from the associated state environmental agency must develop a plan (Total Maximum Daily Load, TMDL) to identify the sources of contamination and the practices that will be implemented to mitigate contamination. In 2016, over 43,000 waters across the U.S. were included on the EPA’s list of impaired waters^13^. Nearly 4,000 water impairments were due to *Escherichia coli*, an EPA-recommended regulatory FIB for recreational freshwaters^14^. Feral swine are known to harbor zoonotic diseases that can be transferred to humans^15,16^. Much of the existing research on pathways of transmission of zoonotic diseases in feral swine is related to transmission to livestock, direct contact with infected animals, consumption of infected farmed or feral swine pork, and contamination of crops^6,16–19^. In contrast, studies of risk from recreational contact with (e.g., swimming, boating, angling) and accidental ingestion of water contaminated by feral swine are largely absent from the literature. Recreational waters are common attractions at many national and state parks, which are also habitat for feral swine. Therefore, potential exposures to feral swine-contaminated water are increasingly common park management concerns.

Congaree National Park (CONG) in South Carolina encompasses more than 26,000 acres (105.22 km) of forested floodplains along the Congaree and Wateree Rivers (https://www.nps.gov/cong). Feral swine populations have rapidly increased at CONG over the last several decades. Researchers have documented significant resource impacts^20,21^ and the park has pursued a comprehensive management strategy to support systematic control efforts^22^. Simultaneously, visitation has grown significantly, increasing over 52% from 95,619 in the year 2000 to 145,929 in 2018^23^. Much of the visitor experience at CONG, including paddling, angling, and hiking, is focused on recreating in and around Cedar Creek. Park staff lack the capacity for systematic FIB monitoring but have recently developed plans that would outline a volunteer-led program^24^.

**Figure 1:**
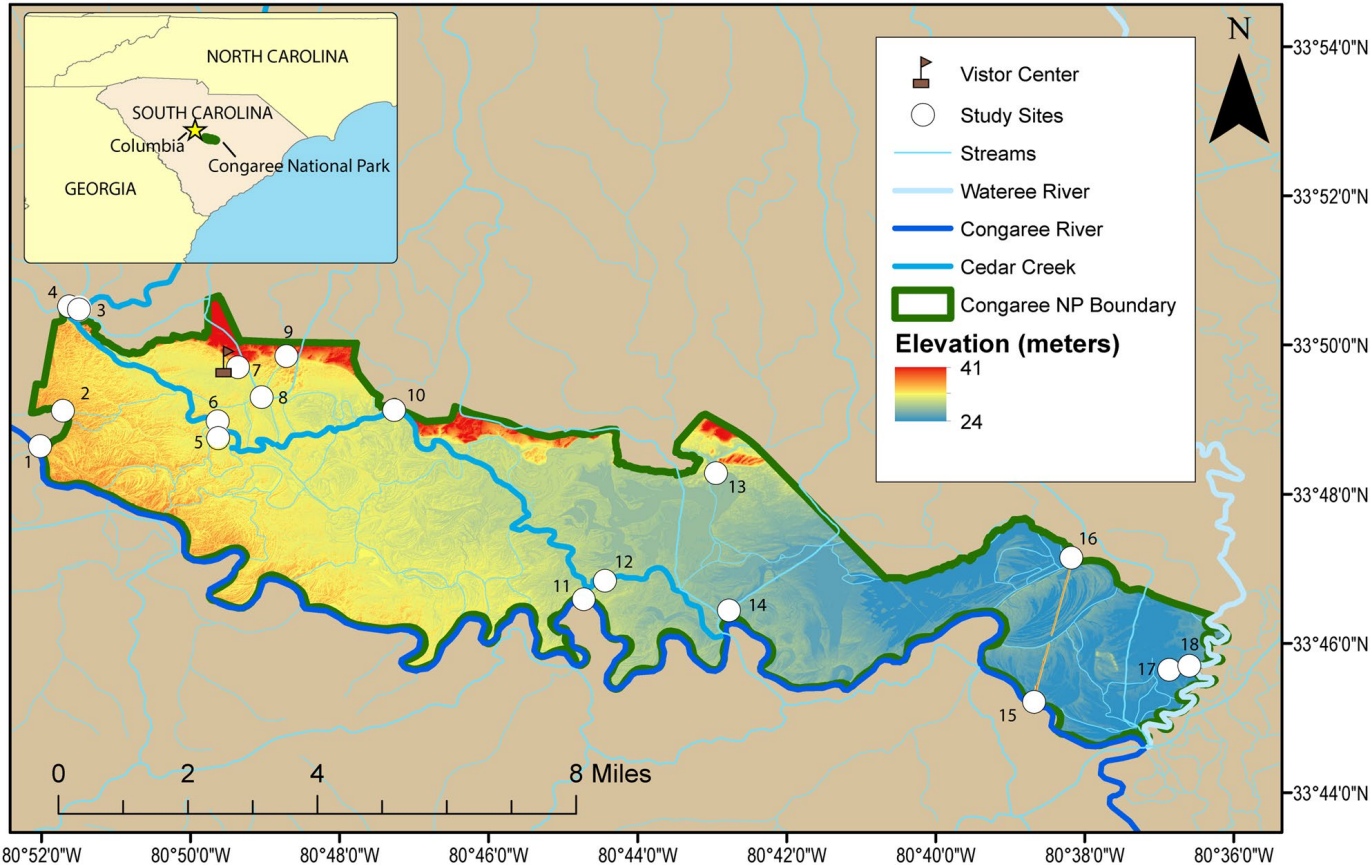
Locations sampled at Congaree National Park. Location names are listed in Table 1. Map created in ArcMap v.10.5.1 (ESRI, https://www.esri.com/).

In 2016, two Cedar Creek surface-water monitoring sites within CONG boundaries were added to the South Carolina Department of Health and Environmental Control 303(d) list of impaired surface waters due to elevated *E. coli* concentrations (Fig. 1, sites 6 and 10)^25^. The capacity for park management to effectively address bacterial contamination, however, has been limited without more data regarding precisely which sources should be targeted for mitigation. While feral swine are one possible source of fecal contamination in CONG, other possible sources include upstream agricultural operations, upstream septic systems, municipal wastewater conveyances, as well as in-park septic systems and native wildlife. Furthermore, the hydrologic setting of CONG facilitates the broad-scale redistribution of water-borne contamination during flood conditions that inundate much of the property. Monitoring FIB alone^24^ can provide a wealth of data for informing specific advisories but cannot discriminate among sources.

Microbial source tracking (MST) is a potential approach for assessing the presence and relative magnitude of enteric bacteria in the environment while identifying the probable animal sources^26–33^. The intestinal systems of different animals such as humans, cattle, deer, or swine are microbiomes with different physiological and dietary signatures and, consequently, distinct enteric bacterial populations. Quantitative polymerase chain reaction (qPCR) is a sensitive molecular technique for detecting target DNA sequences. Bacteria from the order *Bacteroidales* are useful fecal-contaminant indicator microorganisms^34^ because they: (1) are restricted to warm-blooded animals; (2) constitute a relatively significant portion of the bacteria present in mammalian feces; (3) can be highly specific to particular species or taxonomic groups; and (4) are strict anaerobes. In warm waters their DNA may degrade beyond detection within 1 to 3 days^35,36^, which makes them indicative of recent contamination. Taken together, MST results can help identify mitigation targets to include in TMDLs.

In light of the challenges and opportunities at CONG, the objectives of this study were as follows: (1) measure the occurrence and concentrations of MST markers throughout CONG; (2) assess internal and external sources of fecal contamination; (3) assess the frequencies and distributions of detection of a pathogenic marker; and (4) investigate associations between MST marker concentrations and pathogenic marker presence.

## Methods

### Study site

Congaree National Park (CONG) in South Carolina is located in a broad floodplain between the Congaree and Wateree Rivers, and it includes over 11,000 acres (44.52 km^2^) of old-growth forest and over 15,000 acres (60.70 km^2^) of designated federal wilderness^37^. The Congaree River, which forms the southern park boundary (Fig. 1), drains approximately 8,290 mi^2^ (21,476 km^2^) and has varied land use throughout the watershed. Multiple major municipal wastewater systems in the Congaree River basin are within 25 miles upstream from the Park boundary. The state capitol, Columbia, has a population of over 800,000 people in the metropolitan area and is located approximately 20 mi (32.19 km) upstream of the Park. The Wateree River forms a short stretch of the park’s eastern boundary (Fig. 1) and drains over 5,600 mi^2^ (14,500 km^2^). Though not technically part of the park during normal in-bank streamflow conditions, the Congaree and Wateree Rivers (Fig. 1) are additional popular destinations for recreation^37^.

During normal flows on the Congaree and Wateree Rivers, the ecohydrology of the floodplain is dominated by several tributary systems including Cedar Creek, Tom’s Creek, and Dry Branch. Cedar Creek (Fig. 1) is a particularly popular destination for canoeing, kayaking, and fishing. The lower portion of Cedar Creek that flows through the park (between Wise Lake (Site 5) and the Congaree River, Fig. 1, Table 1) is the only waterbody in South Carolina with the designation of Outstanding Natural Resource Water^38^, a state-level designation authorized under the Clean Water Act to recognize water bodies with high ecological importance and exceptional water quality. Land use in the Cedar Creek watershed upstream of the Park is predominantly agricultural (including silviculture, poultry, horses, and farming), with some light industry and rural housing.

**Table 1:** Site name, U.S. Geological Survey Station ID, GPS coordinates, and number of samples collected for sample locations.

Map Number	Site Name	USGS Station ID	Latitude	Longitude	Samples collected
1	Congaree R @ gage	02,169,625	33.81071	− 80.8670	2
2	Cooks Gut	334,907,080,514,300	33.81861	− 80.8619	2
3	Cedar Cr abv Myers Cr	335,031,080,513,800	33.84194	− 80.8606	11
4	Myers Cr abv Cedar Cr	02,169,660	33.84071	− 80.8601	11
5	Wise Lake	334,854,080,493,800	33.81500	− 80.8272	3
6	Cedar Cr @ gage^a^	02,169,672	33.81627	− 80.8273	10
7	Muck Swamp^b^	334,942,080,492,800	33.82833	− 80.8244	2
8	Weston Lake	334,918,080,490,300	33.82167	− 80.8175	3
9	Dry Branch	334,951,080,484,300	33.83083	− 80.8119	11
10	Cedar Cr @ Kingsnake^a^	334,907,080,471,700	33.81877	− 80.7879	11
11	Congaree R abv Cedar Cr	334,636,080,444,300	33.77656	− 80.7454	1
12	Cedar Cr abv Congaree R	334,650,080,442,600	33.78068	− 80.7407	1
13	Toms Cr^a^	334,553,080,425,700	33.80472	− 80.7158	4
14	Stump Gut	334,629,080,424,700	33.77408	− 80.7130	1
15	Congaree R @ SC601	334,513,080,384,100	33.75361	− 80.6447	3
16	Bates Old River	334,709,080,381,100	33.78583	− 80.6364	3
17	Horseshoe Lake	334,539,080,364,800	33.76078	− 80.6132	1
18	Wateree R	334,542,080,363,600	33.76167	− 80.6100	1

^a^On the 2016 South Carolina Department of Health and Environmental Control 303(d) list of impaired surface water for *E. coli*. ^b^Approximate location of onsite septic system.

### Sample collection and processing

One-liter water samples were collected and analyzed from 18 surface-water sites distributed throughout CONG (Fig. 1, Table 1). The sampling strategy was designed to determine if contamination was coming from inside or outside the Park by capturing primary inflows (Congaree River, Cedar Creek, Dry Branch, Myers Creek, Tom’s Creek, Wateree River) at respective upstream and downstream Park boundaries as well as to characterize a cross section of the principal hydrologic features (tributaries, wetland, oxbow lakes; Fig. 1) found within CONG. Eleven sample collection events were conducted between December 2017 and June 2019 to investigate temporal and spatial variability in sources of fecal contamination. Logistical constraints existed that included funding limitations, remote site access, and flooding thus resulting in different frequencies of sampling across study sites. The number of sites sampled during each collection event varied from 4 to 16. Five sites (Sites 3, 4, 6, 9, and 10; Fig. 1) were targeted for repeated sampling (sampled during each sample collection event as conditions allowed) to address temporal variability in sources; the remaining 13 sites were sampled intermittently (one to four times; Table 1) to inform spatial variability in sources.

Grab samples for molecular analysis were stored in coolers on ice until they were transferred to a refrigerator at the laboratory until processing, which occurred within 48 h of sample collection. Samples were filtered on a vacuum manifold through a 47-mm polycarbonate filter with 0.4-µm pore size (Millipore, Bedford, MA, USA). Samples were filtered in 100-mL aliquots or to the point of the filter clogging (minimum volume 20-mL^39^). Six filter replicates were collected per sample that included two filters that were enriched for *E. coli* with modified mTEC agar following the incubation protocol in EPA Method 1603^40^ to enhance detection of shiga-toxin producing *E. coli* STEC^41,42^ and four unenriched filters. Two unenriched filters and one enriched filter were processed for molecular analysis. The remaining filter replicates were retained as backups for use if needed. All filter replicates were stored at − 80 °C until DNA extraction. Sterile de-ionized water (sterile DI; autoclaved 15 PSI, 121 °C for 15 min per 1-L of sterile DI^43^) controls were filtered in conjunction with each sample event to determine presence of potential contamination during sample collection and/or laboratory processing and were treated identically to filtered samples in downstream processing.

### Citizen science Escherichia coli analysis

Beginning in October 2018, select duplicate water samples were collected for *E. coli* analysis at the CONG Old-Growth Bottomland Forest Research and Education Center as part of a Citizen Science methods development program by CONG personnel as described in McCarthy^24^. Samples were processed within 6 h of collection and kept on wet ice during the time between collection and processing. Each sample was screened at three dilutions to ensure accurate and quantifiable results. Samples were tested for *E. coli* concentrations and enumerated using the most probable number (MPN) method with IDEXX Colilert-18 (IDEXX Laboratories, Inc. Westbrook, ME) for enumerating *E. coli*. A laboratory negative control (sterile DI) was analyzed alongside samples for each sample event to test for contamination in reagents or contamination potentially introduced during sample processing. Resulting data were used in a post-hoc analysis to investigate relationships between *E. coli* MPN and MST marker concentrations.

### DNA extraction and quantitative PCR

DNA was extracted using the Qiagen DNeasy PowerLyzer PowerSoil DNA Isolation Kit (Qiagen; Germantown, MD)^44^ following manufacturer’s instructions. We did not explicitly test the efficiency of the DNA extractions; however, this isolation kit has been shown to efficiently extract *Bacteroides* and *E. coli* DNA from stool samples^45^. Samples were tested for PCR inhibition following Bradshaw, et al.^46^ using the Sketa22 assay^47^. Samples were screened with probe-based quantitative PCR (qPCR) MST markers for humans (HF183^48^, Table 2), ruminants (Rum2Bac^49^, Table 2), chickens (CL^50^, Table 2), and swine (Pig2Bac^49^, Table 2). Samples that tested positive for ruminants were also screened for cows (CowM3^51^, Table 2). PCR efficiency ranged from 82 to 102%^39^. Enriched samples were screened for *stx2*^52^ (Table 2). Pig2Bac has been found to be highly sensitive, but the MST marker has been found to occur in dog feces, human feces, and septage^32^. To test for possible cross amplification of Pig2Bac with human contamination, we calculated Spearman’s *ρ* for the correlation between concentrations of HF183 and Pig2Bac. A significant correlation between Pig2Bac and HF183 could indicate that Pig2Bac concentrations included detection of human contamination.

**Table 2:** Quantitative PCR microbial source tracking markers and the attribution of the source if detected in a sample.

Marker name	Primer and probe sequences 5′ → 3’	Product length (base pairs)	Attribution	Target organism and gene	References
**HF183**		126	Human	*Bacteroides* 16 s rRNA	^44^
HF183	ATCATGAGTTCACATGTCCG				
BacR287	CTTCCTCTCAGAACCCCTATCC				
BacP234MGB	FAM-CTAATGGAACGCATCCC-MGB				
**Rum2Bac**		99	Ruminant^a^	Bacteroidales 16 s rRNA	^45^
BacB2-590F	ACAGCCCGCGATTGATACTGGTAA				
Bac708Rm	CAATCGGAGTTCTTCGTGAT				
BacB2-626P	FAM-ATGAGGTGGATGGAATTCGTGGTGT-TAMRA				
**CL**		78	Chicken litter	*Brevibacterium* sp*.* 16 s rRNA	^46^
CLF	CCCGGGAAACTGGGTCTAAT				
CLR	CCATCCCCAATCGAAAAACTT				
CLP	6FAM-CCGGATACGACCATCTGCCGCA-TAMRA				
**Pig2Bac**		116	Feral swine^b^	Bacteroidales 16 s rRNA	^49^
Bac41F	GCATGAATTTAGCTTGCTAAATTTGAT				
Bac163Rm	ACCTCATACGGTATTAATCCGC				
Bac113MGB	FAM-TCCACGGGATAGCC-NFQ-MGB				
**CowM3**		122	Cow	Bacteroidales Sialic acid-specific 9-O-acetylesterase secretory protein homolog	^47^
CowM3F	CCTCTAATGGAAAATGGATGGTATCT				
CowM3R	CCATACTTCGCCTGCTAATACCTT				
CowM3Probe	FAM-TTATGCATTGAGCATCGAGGCC-TAMRA				
**stx2**		65	Shiga toxin- producing *E. coli*	*Escherichia coli stx2*	^48^
*stx2*F	ACGGACAGCAGTTATACCACTCT				
*stx2*R	CTGATTTGCATTCCGGAACGT				
*stx2*P	FAM-CCAGCGCTGCGACACG-NFQ				

^a^Rum2Bac detection without CowM3 detection was interpreted as white-tailed deer, ^b^ no large hog operations are located in the contributing watersheds.

DNA from two filter replicates per sample were screened for each marker with two qPCR replicates for a total of four qPCR replicates per sample per marker. Reactions were carried out in 96-well qPCR plates in 20-µL reaction volumes. Final concentrations of reagents in the assays were 1-µM forward and reverse primers, 80-nM 6-carboxy-fluorescein FAM- labeled TaqMan probe, 0.02-mg/mL BSA (Life Technologies), 1 × DNA TaqMan Fast Universal PCR Master Mix (Life Technologies), and 4-µL of template (i.e., genomic DNA, nanopore water as a no template control, or MST marker standard concentrations of 10 to 10^6^ copies). Synthetic gene fragments with the marker insert sequences were used to create the standard curves for all MST markers and as a qPCR positive template control for *stx2*. Microbial source tracking markers with low specificity can reduce confidence that a detection is indicative of contamination from the targeted host species. To reduce the likelihood of spurious detection of an MST marker from a non-target host species, samples were considered absent for a marker when amplification occurred in zero or one of the four qPCR replicates. Samples absent for an MST marker were assigned an estimated concentration of 0 gene copies. The *stx2* assay was analyzed as presence/absence where amplification in one or both qPCR replicates was considered evidence of presence. With the exception of the standard curve qPCR replicates for MST markers and positive template control reactions for the *stx2* qPCR assay, no procedural positive controls were included during sample processing. Therefore, the influence of variable enrichment efficiencies for *E. coli* or DNA extraction efficiencies among samples on MST marker concentration or *stx2* presence/absence results cannot be ruled out. Across all MST markers, R^2^ values for standard curves ranged from 0.987 to 0.999, PCR amplification efficiencies ranged from 82 to 102% for all markers, and slopes ranged from -3.85 to -3.27. The limit of quantification (LOQ) for each MST marker was determined as the lowest standard curve copy number with a coefficient of variation (CV = 100*σ/µ; where σ is the standard deviation, and µ is the mean number of estimated gene copies) less than 35%. Back calculated gene copy estimates were based on the equation X_0_ = E_AMP_(b-C_T_) where X_0_ is the initial number of target copies in the qPCR; E_AMP_ is the exponential amplification value, which is 1 + the amplification efficiency (e.g., if the amplification efficiency is 0.94, E_AMP_ = 1.94); b is the y-intercept of the standard curve; and C_T_ is the cycle number when the amplification curve crosses the threshold line that distinguishes fluorescence intensity of a reaction from background levels.

### Statistical analysis

A statistical technique similar to McKee, et al.^33^ was used to account for error in qPCR estimates below LOQ. If a qPCR estimated gene copy number was greater than 0 and below LOQ, a random number was selected from a normal distribution centered at the qPCR estimated copy number. Randomly selected negative values were set to 0 and randomly selected values above LOQ were set to the LOQ. To determine the standard distribution of the error estimates for each marker, a trendline was created with the standard deviations of the back-calculated gene copy number estimates for the standard curve gene copy numbers at and below LOQ (Table A.1). An additional data point at the origin (0, 0) served as a value for the no template controls. The equation for that trendline was used to calculate the standard deviation for each randomly selected number, which was a function of the qPCR estimated gene copy number. Randomly selected numbers were treated the same as qPCR measured concentrations above the LOQ for statistical analysis. All analyses were conducted in JMP v 14.2.0 (SAS Institute Inc.). Recursive partitioning was conducted to determine the MST marker and concentration that best predicted *stx2* detection and *E. coli* density splits. Recursive partitioning is a nonparametric data-mining analysis that splits the response variable into two categories based on an explanatory variable cutting value. The cutting value is determined with a partition algorithm that finds the explanatory variable split that maximizes the difference in the response frequencies between the two nodes of the split. Spearman’s *ρ* were used to assess correlative relationships between MST marker concentrations and *E. coli* MPN. To account for multiple comparisons of the same data, we used p < 0.01 to indicate statistical significance for all Spearman’s rank correlations.

## Results

HF183 and Pig2Bac were detected at 14 sites, Rum2Bac at 2 sites, and *stx2* at 7 sites. Pig2Bac was detected in 37 samples (46%), which was more frequent than HF183 (21 samples, 26%), Rum2Bac (4 samples, 5%), and *stx2* (15 samples, 19%). Of the 324 qPCR replicates per MST marker, many qPCR replicate concentrations were less than LOQ and therefore estimated as described above (HF183: 74 qPCR replicates from 24 samples; Pig2Bac: 67 qPCR replicates from 25 samples; Rum2Bac: 16 qPCR replicates from four samples; Table A.2). Spearman’s rank correlation between HF183 and Pig2Bac was not significant (*ρ* = 0.445, p = 0.026, Table 3) and HF183 was detected in only 15 of 37 samples that were positive for Pig2Bac, suggesting limited influence of human contamination on Pig2Bac concentrations.

**Table 3:** Spearman’s rank correlations (*ρ*) among MST markers and *E. coli* MPN.

	HF183	Pig2Bac	Rum2Bac	*E. coli* MPN
HF183		0.445	-0.059	0.146
Pig2Bac	0.026		0.219	0.437
Rum2Bac	0.781	0.293		0.478
*E. coli* MPN	0.485	0.029	0.016	

Values above the diagonal are Spearman’s *ρ*. Values below the diagonal are p-values. Statistical significance was determined when p < 0.01.

### Spatial and temporal variability in sources throughout the Park and at Cedar Creek

**Figure 2:**
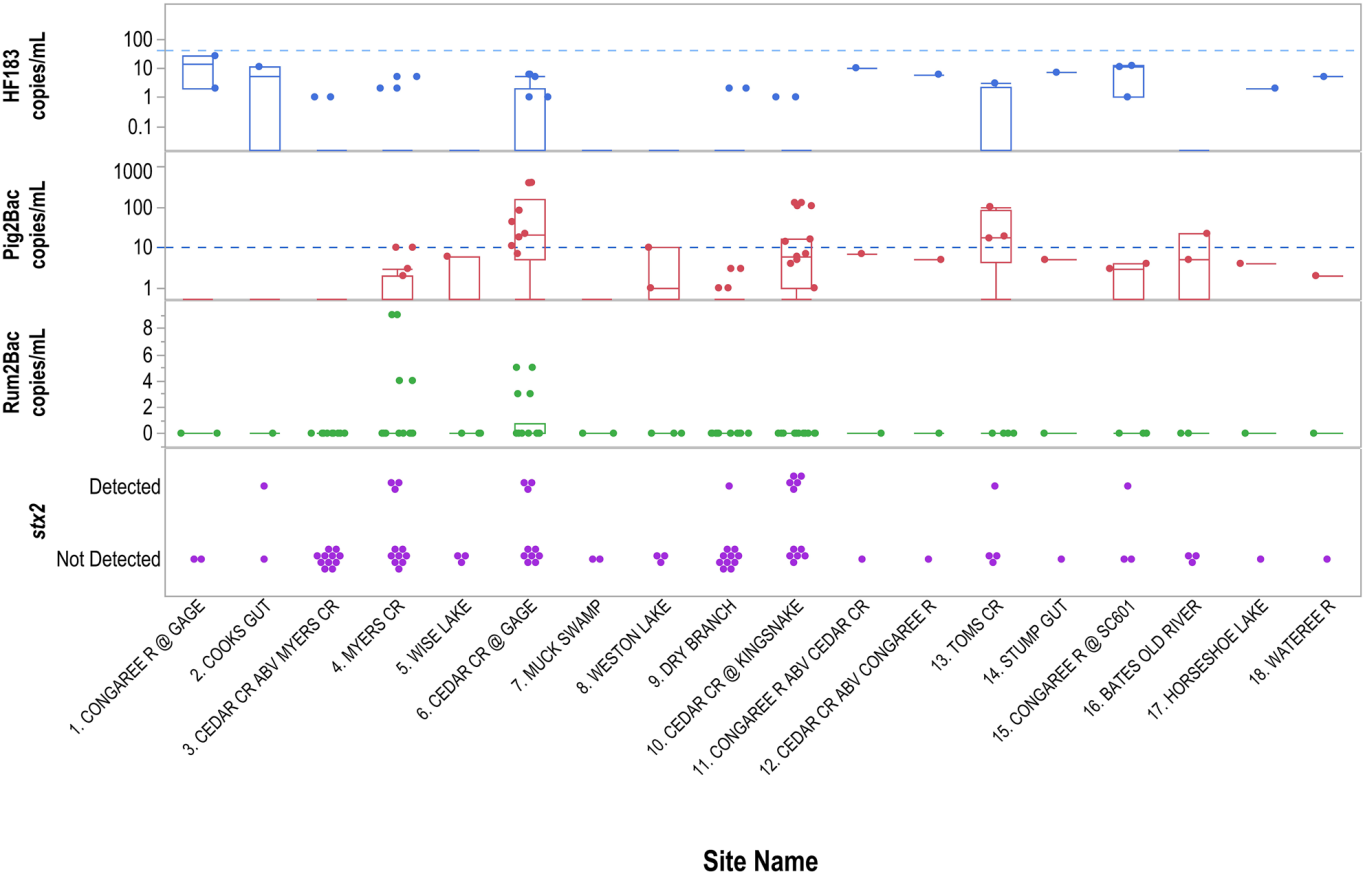
Quantitative PCR (qPCR) microbial source tracking marker concentrations (gene copies per mL) for humans (HF183), swine (Pig2Bac), ruminants (Rum2Bac), and qPCR replicate detections of Shiga toxin gene marker (stx2) in water samples per site throughout Congaree National Park. Dots represent individual samples. Dashed blue lines indicate the limit of quantification (LOQ). Box indicates first and third quartiles. The upper and lower whiskers extend to the largest and smallest values within 150% of the inter-quartile range (non-detections for HF183 and Pig2Bac not shown on log scale).

**Figure 3:**
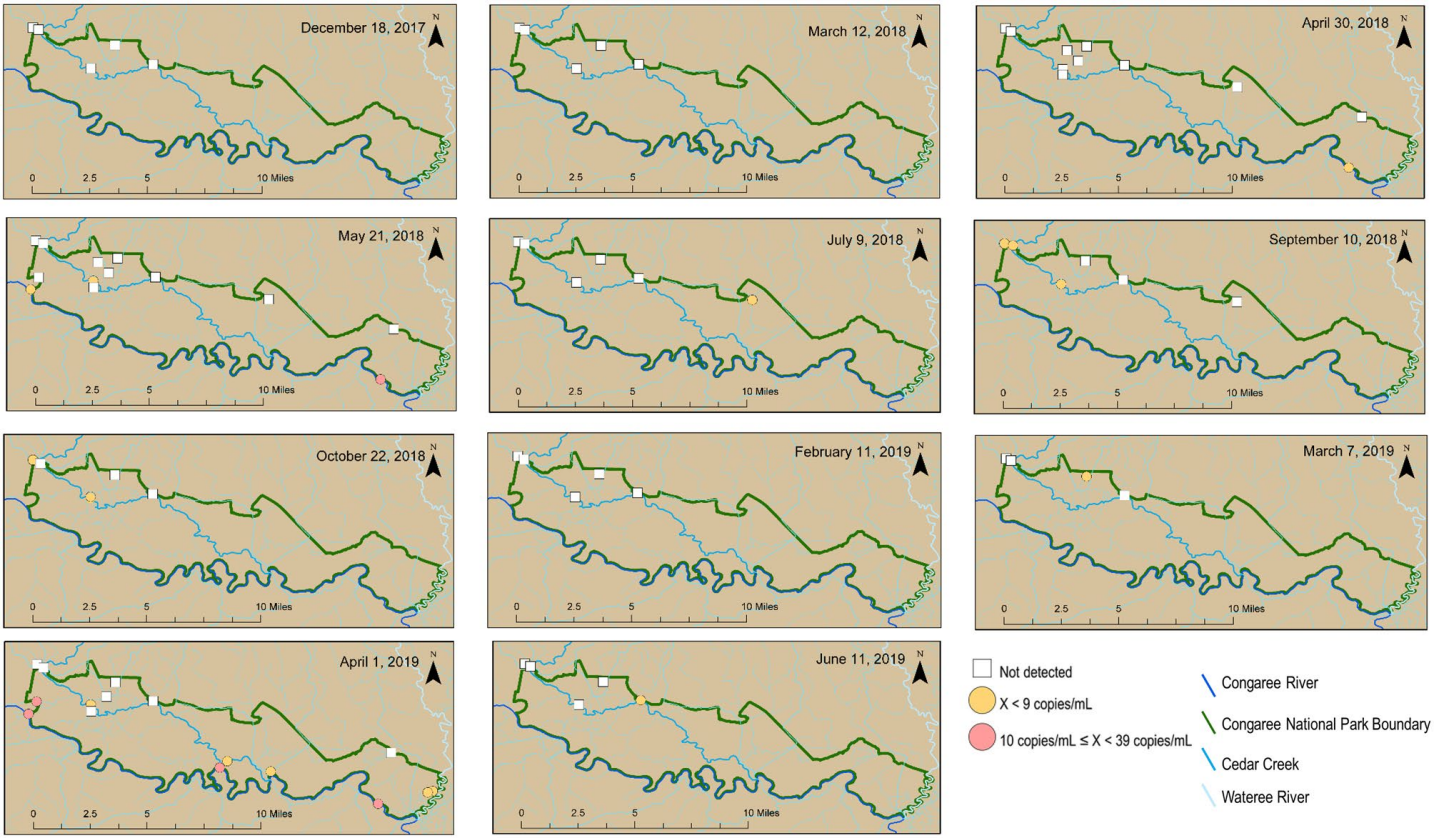
HF183 concentrations throughout and surrounding Congaree National Park for samples collected between December 18, 2017 and June 11, 2019. Maps created in ArcMap v.10.5.1 (ESRI, https://www.esri.com/).

**Figure 4:**
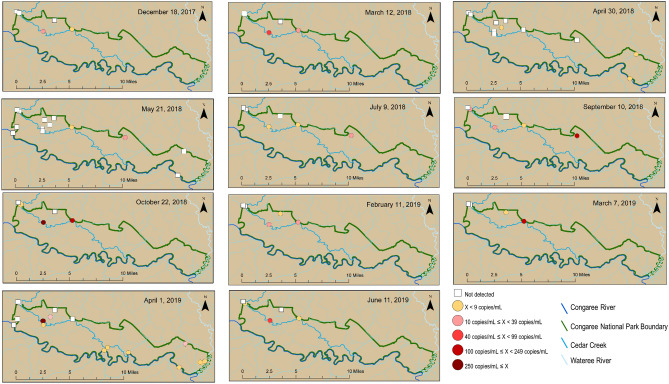
Pig2Bac concentrations throughout and surrounding Congaree National Park for samples collected between December 18, 2017 and June 11, 2019. Maps created in ArcMap v.10.5.1 (ESRI, https://www.esri.com/).

**Figure 5:**
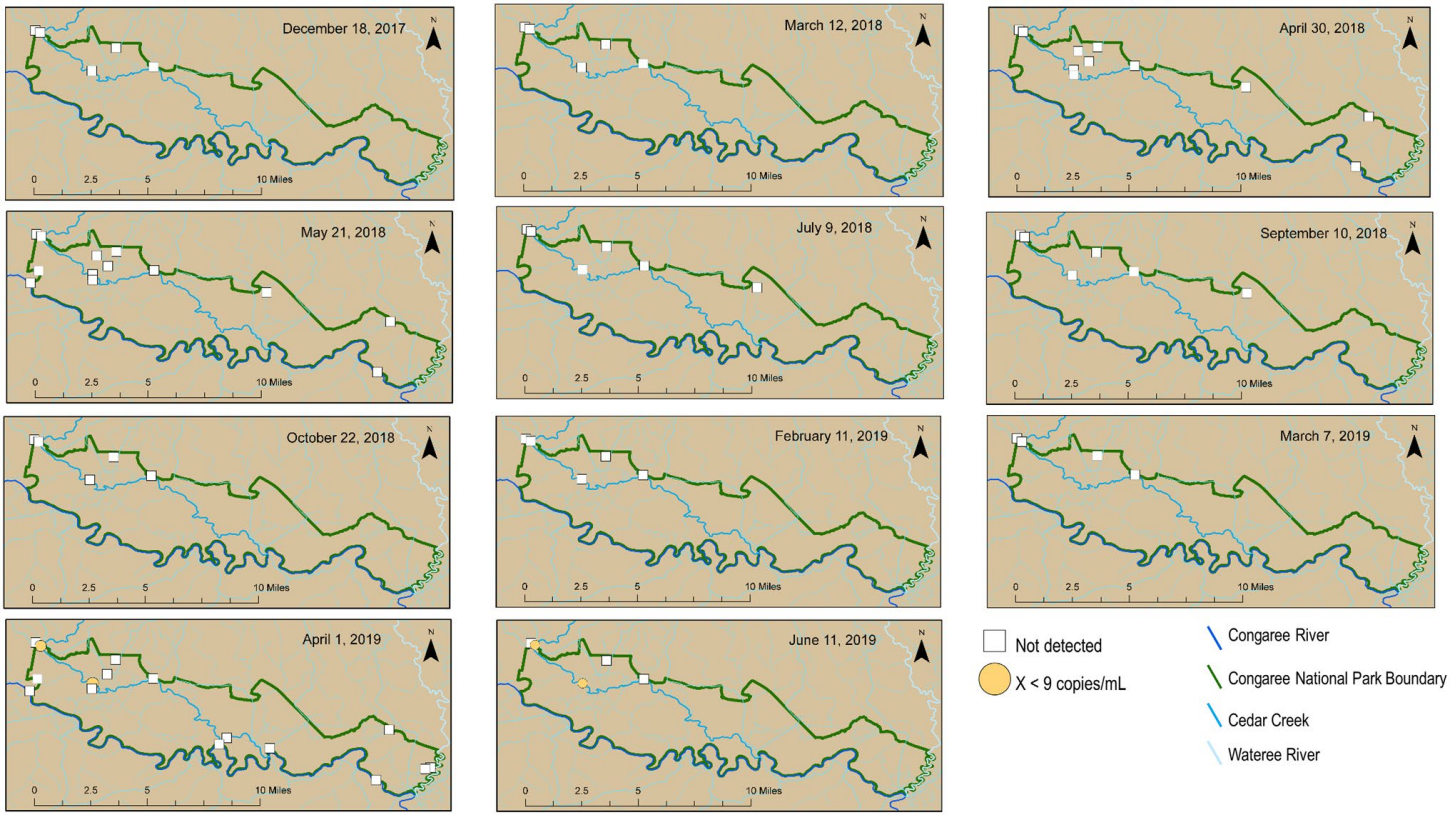
Rum2Bac concentrations within and around Congaree National Park for samples collected between December 18, 2017 and June 11, 2019. Maps created in ArcMap v.10.5.1 (ESRI, https://www.esri.com/).

**Figure 6:**
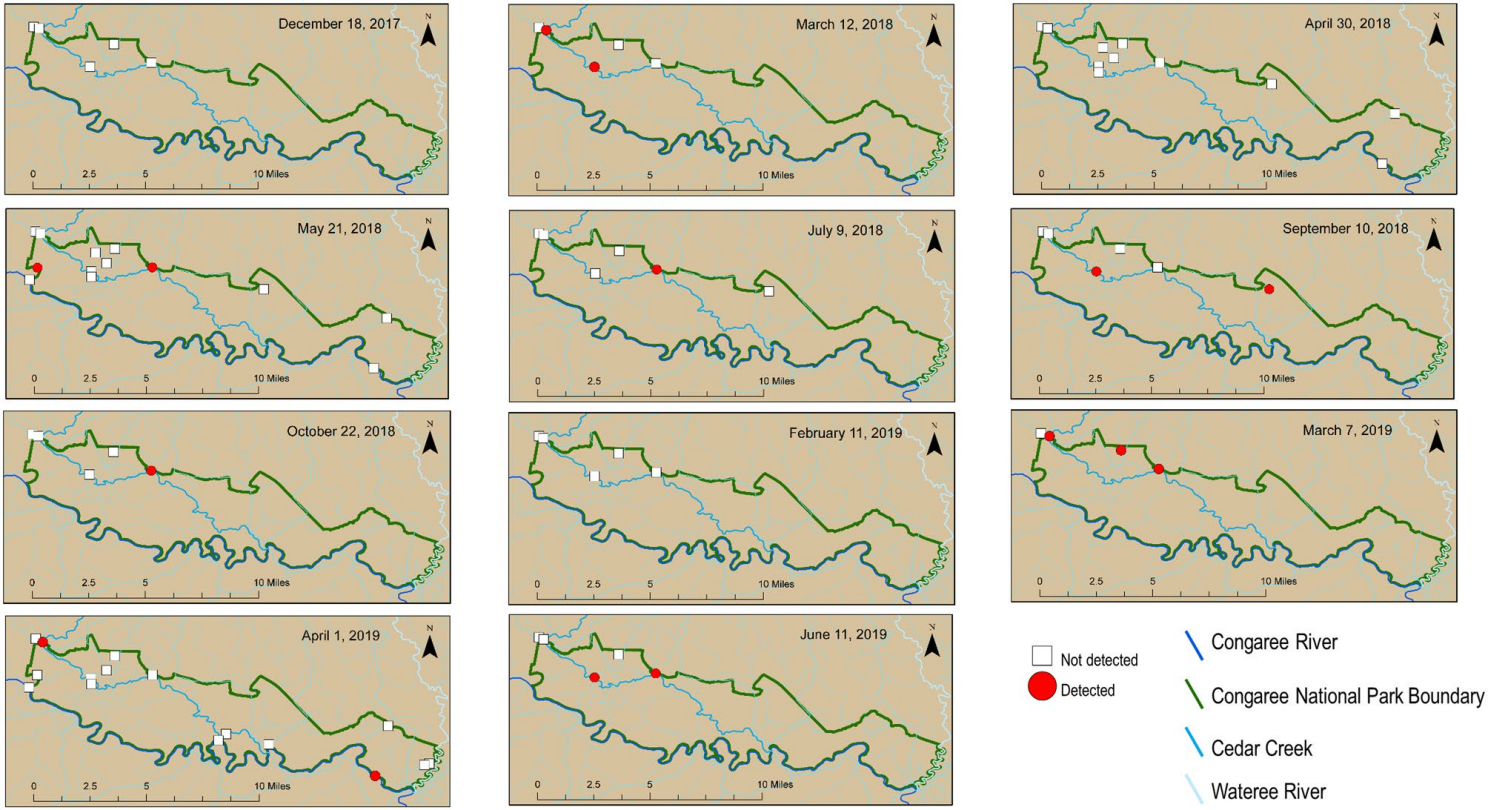
*stx2* marker detections within and surrounding Congaree National Park for samples collected between December 18, 2017 and June 11, 2019. Maps created in ArcMap v.10.5.1 (ESRI, https://www.esri.com/).

HF183 and Pig2Bac were detected broadly across the study sites (Figs. 2, 3 and 4), with detections at sites inside, outside, and on the boundary of CONG (Figs. 3, 4). The highest concentrations of HF183 detected were on the Congaree River boundary waters (Site 1, Congaree R @ gage: 26 copies/mL; and Site 15, Congaree R @ SC601: 12 copies/mL, Fig. 2, 3). HF183 was the only marker detected at all four sites along Cedar Creek, with a maximum detected concentration of 6 copies/mL detected at both Cedar Cr @ gage and Cedar Cr abv Congaree R (Sites 6 and 12, respectively; Figs. 2, 3). The highest concentrations of Pig2Bac detected across all sites were from samples collected from two of the three study sites on the 2016 South Carolina 303(d) list: Site 6, Cedar Cr @ gage 398 copies/mL; and Site 10, Cedar Cr. @ Kingsnake 127 copies/mL (Figs. 2, 4). Rum2Bac was detected at two sites, one immediately upstream of the Park boundary (Site 4, Myers Cr abv Cedar Cr) and one within the Park on Cedar Cr @ gage (Site 6, Fig. 5). The *stx2* marker was detected at boundary water sites, as well as sites within and upstream of CONG (Fig. 6). Of the internal sites, *stx2* was detected most frequently in samples from Cedar Cr @ Kingsnake (Site 10, five of 11 samples, Fig. 6).

Temporally, Pig2Bac was the most consistent marker to be detected, because it was present in one or more samples for 10 of 11 sample events (Fig. 4). HF183 was detected at one or more sites for 6 of 11 sample events and Rum2Bac was only detected in samples from the last two sample events (Figs. 3 and 5). The *stx2* marker was detected in samples from at least one site for 8 of 11 sample events (Fig. 6).

### MST marker predictions of shiga-toxin producing E. coli presence and E. coli MPN

None of the three MST markers were strong predictors of *stx2* detection. Recursive partitioning indicated that the best predictor of *stx2* detection across all samples was Pig2Bac concentrations ≥ 43 copies/mL (R^2^ = 0.135). The Pig2Bac split correctly identified 5 of 15 samples with *stx2* detections. Samples with Pig2Bac concentrations ≥ 43 copies/mL were associated with an average 51% greater probability of *stx2* detection compared to only a 14% probability of *stx2* detection in samples with Pig2Bac concentrations < 43 copies/mL. All samples with *stx2* detections that were positive for HF183 were also positive for Pig2Bac, whereas seven samples with *stx2* detections were positive for Pig2Bac but not HF183. Three of the four samples with *stx2* detections that were not positive for either HF183 or Pig2Bac were from Myers Creek. *stx2* was only detected in two samples for which Rum2Bac was also detected.

Posthoc analysis with culturable *E. coli* data from citizen science collected samples (Table A.2) also indicated that Pig2Bac concentrations ≥ 43 copies/mL were the best recursive partitioning split for *E. coli* MPN (R^2^ = 0.331, root mean square error = 137.6, AICc = 324.3). When Pig2Bac concentrations were ≥ 43 copies/mL, *E. coli* concentrations had a mean of 357 MPN (± 195 standard deviation). In contrast, mean *E. coli* concentration for samples with Pig2Bac concentrations < 43 copies/mL was 115 MPN (± 130), indicating an average increase in *E. coli* MPN of 242 in samples containing ≥ 43 copies/mL of Pig2Bac. No significant correlations were detected between MST markers and *E. coli* MPN (Table 3).

## Discussion

Results of this study suggest that humans are an intermittent source of bacterial contamination and swine are primary contributors of fecal contamination in CONG. Infrequent detection of the ruminant marker and no detections of the cow or poultry marker suggest that wildlife, cattle, and poultry operations were not major contributors of contamination in the Park at the time of sample collection.

### Internal versus external sources of contamination

Human MST marker data are consistent with upstream inputs at park boundaries as well as some backcountry locations, but show no evidence that the park’s primary visitor center septic system is a major source of fecal contamination in recreational waters within CONG. Feral swine markers, by contrast, are consistent with inputs from feral swine internal to CONG. Possible sources of contamination from outside the Park include failing septic systems and leaking wastewater conveyances. Sources of human contamination from within the park could include on-site septic systems as well as local backcountry contributions from hikers and campers. HF183 was detected at all four Cedar Creek sites, however, concentrations were never at levels above 10 copies/mL and all were below the LOQ. The highest concentrations of the human marker were observed from Congaree River samples, consistent with external municipal or residential wastewater conveyances as a potential source. Human contamination was not detected at the Muck Swamp site, located adjacent to the Park’s Visitor Center septic system, suggesting that the on-site septic system was likely not a major net source of bacterial contamination during this study. It is possible that lack of septic contamination here could be related to its filtering through organic rich soils in Muck Swamp, as these types of tannin-rich settings have been noted to have natural anti-bacterial properties^54^. Another previously suggested possible source of human waste contamination within the Park is direct local inputs of contamination associated with visitation in the backcountry. Contaminants indicative of anthropogenic contamination have been found in water samples from non-stream sites at CONG, including samples from Muck Swamp, Wise Lake, and Weston Lake, under non-flood conditions (i.e., in the absence of a surface-water connection to extra-Park contaminant sources on the Congaree and Wateree Rivers or tributaries)^55^. In contrast, under flood conditions, interior Park locations can be hydrologically connected to the Congaree and Wateree Rivers and the associated external human wastewater sources. Two U.S. Geological Survey real-time gaging stations on the Congaree River (Congaree R @ Gage, site 1; Fig. 1) and Cedar Creek (Cedar Cr @ Gage, site 6; Fig. 1) are used to inform the public when the Park is under flood conditions based on gage height (15 feet or greater at Congaree R @ Gage, 8 feet or greater for Cedar Cr @ Gage; www.nps.gov/cong/planyourvisit/conditions.htm, accessed Feb. 28, 2020). Data from April 1, 2019 (waterdata.usgs.gov/, accessed Feb. 28, 2020) indicate CONG was not under flood conditions when the human MST marker was detected at Stump Gut, Horseshoe Lake, and Cedar Creek @ Gage suggesting contamination may have come from internal inputs. Other detections of human contamination at the Cedar Cr. @ Gage site were concurrent with upstream detections at the Cedar Creek upstream Park boundary sites at Cedar Cr. @ Myers and Myers Cr., consistent with downstream transport from external sources during non-flood conditions. All other detections of human contamination were at boundary water sites, or sites proximal to the boundary and downstream from potential external sources.

Feral swine are well documented throughout the southeastern U.S. and particularly in the study region of the Coastal Plain in South Carolina^5^. The lack of hog farms within the Congaree River watershed indicates feral swine as the main source of the swine MST marker in CONG. Further, while a comparison of swine marker loads (concentration per unit time) would be necessary for verification, higher swine MST marker concentrations at Cedar Creek sites within the Park compared to sites at the upstream boundary of the Park suggests swine contamination is predominantly, but not exclusively, occurring within CONG.

The home ranges of feral swine follow similar patterns to other mammals and tend to be larger for males than females. Feral swine home ranges can vary annually and seasonally and are generally smaller when food sources are more abundant^56,57^. Previous studies on feral swine home ranges in South Carolina have indicated that in coastal marshes, the average home range sizes were 2.26 km^2^ for males and 1.81 km^2^ for females^58^ and 3.89 km^2^ on average for males and females in the Piedmont^59^. In-house data from Park files and partner research suggest that local swine can move up to four miles per day and swim the Congaree River multiple times per day, indicating that detections of the swine MST marker external to CONG could be from feral swine the home ranges of which include the Park.

There are a couple limitations to the results from this study. One limitation is that the methods did not test for markers from the American alligator (*Alligator mississipianensis*). Research suggests that crocodilian feces are exceptionally rich in FIB^60^, although limited research has been conducted on pathogenic *E. coli* in reptiles^61^. Research at the Park suggests that the local alligator populations are low density and transient, but they could be disproportionately represented in some samples. Most alligators, however, are concentrated along the Congaree and Wateree Rivers at the southeast end of the park and well away from the Cedar Creek locations central to this analysis. Additionally, analysis for specific markers for turkey and other avian sources were not conducted, and these animals can represent a significant portion of the non-swine and non-deer megafauna in the Park. A second limitation to the study is that we cannot rule out non-specific amplification of the swine marker as a factor in the frequency of detection and estimated concentrations of the swine marker over the course of the study. We did not detect a correlation between the human and swine marker concentrations, which we interpreted as evidence that non-specific amplification from the swine marker was not occurring when human fecal contamination was present. However, the lack of correlation may have been caused by differences in stability of the signal between the human and swine markers due to factors such as differences in decay rates among markers from different sources^62^. Differences in environmental persistence among MST markers and markers from different sources may also explain why we did not detect a significant association between any of the MST markers and the pathogenic *E. coli* marker.

### Human health risk from exposure to water with fecal contamination

We did not find evidence of a strong association between sources of fecal contamination and presence of STEC. Possible methodological explanations for the lack of a strong association between *stx2* presence and any of the MST markers include variable efficiencies in bacterial recovery from environmental samples, different DNA extraction efficiencies among samples, and suboptimal incubation conditions for STEC. Unlike most other fecal coliforms, which are characterized by their ability to grow at 44.5–45.5 °C, studies have suggested that *E. coli* O157:H7, the most common serotype of STEC to cause infection^63,64^, does not grow well at temperatures above 41 °C^64,65^. However, Duris, et al.^42^ detected STEC in more than 50% of river water samples from Michigan and Indiana using a similar incubation protocol as used in the study herein. Results from our study and Duris, et al.^42^ indicate that incubating enriched samples at 44.5 °C does not inherently prevent detection of STEC, although presence may be underestimated.

Recursive partitioning of *E. coli* indicated that swine MST marker levels above 43 copies/mL were associated with *E. coli* levels above the EPA beach action value (BAV) for recreational freshwaters of 235 colony forming units/100 mL (CFU/100 mL; conversion between CFU to MPN is 1:1). This suggests that when Pig2Bac levels are above a certain threshold, FIB levels are likely to exceed BAV levels. The *E. coli* BAV for recreational freshwaters is based on investigations of the frequency of gastro-intestinal (GI) illness in swimmers exposed to water downstream of a sewage treatment facility and thus primarily exposed to human contamination^14^. Research suggests that, in general, exposure to human fecal contamination may be a greater human-health risk than exposure to fecal contamination from non-human sources^66–68^. This is likely due in part to the host specificity of many viruses^69^ that are the etiologic agents most frequently responsible for human illness associated with exposure to recreational waters^70^. Therefore, the health risk from exposure to recreational freshwater contaminated by non-human sources may not be accurately represented by the BAV. Additionally, the BAV was developed to indicate the likelihood of illness from recreational exposure to contaminated waters. Feral swine zoonoses caused by exposure to contaminated freshwater may present increased variability in the severity of illness that is not represented by the BAV.

Studies suggest that exposure to fecal contamination from domestic pigs presents a lower health risk than exposure to human fecal contamination^66,71^. Health risks from exposure to recreational waters with FIB densities of 35 CFU/100 mL enterococci and 126 CFU/100 mL *E. coli* from domestic pig fecal contamination were estimated to be substantially lower than the risk from exposure to human sources, with the median risk from domestic pigs two orders of magnitude lower than the human-based benchmarks^66^. Based on these findings, a new water-quality benchmark for domestic pigs should not be ruled out^66^.

If health risks from exposure to domestic pig and feral swine fecal contamination are similar, the frequency of detection of the feral swine MST marker in CONG relative to the human MST marker may indicate the health risk is lower than would be predicted if humans were the predominant source of contamination. However, illness rates from exposure to feral swine fecal contamination may differ from illness rates due to exposure to domestic pig contamination. Feral swine and domestic pigs have been found to serve as reservoirs to different enteropathogenic bacterial strains^72^, suggesting that health risks from exposure to domestic pig fecal contamination may not accurately represent the risk from exposure to feral swine. Feral swine are known to be hosts for numerous zoonotic pathogens, including *Mycobacterium avium* complex^73^, which can cause respiratory illness and be transmitted through recreational waters^74^. The application of quantitative microbial risk assessments^75^ may be useful for predicting the likelihood of illness from zoonotic pathogens in recreational waters with feral swine fecal contamination. Further research is needed to determine the human health risks from exposure to feral swine contamination and to identify other locations where human exposure to feral swine fecal contamination is likely to occur from contact with contaminated recreational waters.

### Regulatory implications of wildlife contamination in impaired recreational waters

Study data suggesting significant wildlife inputs (relative to humans) have potential regulatory implications. Current (2020) EPA Recreational Water Quality Criteria (RWQC)^14^ acknowledge the potential for differences in human health risks from exposure to recreational waters contaminated by human versus non-human sources but do not provide water-quality standard recommendations that take sources of fecal contamination into account. Instead, the 2012 RWQC describes methods that can be used to develop site-specific standards for states that desire to address the variability in human health risks associated with different sources of fecal contamination. In Florida, MST was used to assess sources of fecal contamination in a wildlife conservation-managed watershed that was included on the 2010 Florida Department of Environmental Protection 303(d) list of impaired waters for FIB^76^. Results from the MST analysis suggested that birds, not humans, were the main source of contamination^76^. A natural source exclusion status was obtained for the water body, which was subsequently removed from the Florida 303(d) list. At least 13 other states have natural resource—or “natural conditions”— exclusions in their administrative code, although the conditions for these exceptions and how they are applied can vary by state^77^. Intermittent detection of human contamination in South Carolina 303(d) listed impaired recreational waters in CONG may preclude these sites from a natural source exclusion. Further research would be necessary to determine if a natural resource exclusion would be appropriate or possible for the impaired streams in CONG.

## Supplementary Information

Supplementary Information

## Data Availability

Quantitative PCR data for this study are available in the associated USGS data release^39^ in the USGS ScienceBase-Catalog, which is the authoritative repository of these data.
