# Supplementary material for: Feral swine as sources of fecal contamination in recreational waters
Source: Sci Rep. 2021 Feb 18;11:4212. doi: 10.1038/s41598-021-83798-6 (PMC7893155; doi:10.1038/s41598-021-83798-6)
Supplement: Supplementary file 1 — Supplementary Information [file 41598_2021_83798_MOESM1_ESM.pdf]

Feral swine as sources of fecal contamination in recreational waters

Anna M. McKee, Paul M. Bradley, David Shelley, Shea McCarthy, and Marirosa Molina

Table A.1. Back-calculated gene copy numbers for HF183, Rum2Bac, and Pig2Bac to determine the standard distribution of the error estimates for each marker. The standard deviation of the back-calculated values for the standard curve gene copy numbers at and below the limit of quantification (LOQ), and an additional data point at the origin (0, 0) that represented no template controls, were used to create a trendline. The equation for that trendline was used to calculate the standard deviation for the randomly generated MST marker concentrations below the LOQ.

MST Marker	qPCR standard gene copy quantity	Back-calculated gene copy quantity
HF183	10	6
HF183	10	8
HF183	10	12
HF183	10	11
HF183	10	12
HF183	10	15
HF183	10	21
HF183	10	5
HF183	10	13
HF183	10	8
HF183	10	7
HF183	10	8
HF183	10	7
HF183	10	12
HF183	10	14
HF183	10	10
HF183	40	31
HF183	40	52
HF183	40	35
HF183	40	36
HF183	40	25
HF183	40	43
HF183	40	23
HF183	40	42
HF183	40	14
HF183	40	31
HF183	40	21
HF183	40	42
HF183	40	34
HF183	40	47
HF183	40	59
HF183	40	36
HF183	40	26
Rum2Bac	10	17
Rum2Bac	10	10
Rum2Bac	10	5
Rum2Bac	10	14
Rum2Bac	10	6
Rum2Bac	10	21
Rum2Bac	10	6
Rum2Bac	10	5
Rum2Bac	10	14

Rum2Bac	10	12
Rum2Bac	10	10
Rum2Bac	10	12
Rum2Bac	10	5
Rum2Bac	10	15
Rum2Bac	10	8
Rum2Bac	10	6
Rum2Bac	10	12
Rum2Bac	40	33
Rum2Bac	40	29
Rum2Bac	40	36
Rum2Bac	40	40
Rum2Bac	40	40
Rum2Bac	40	22
Rum2Bac	40	31
Rum2Bac	40	43
Rum2Bac	40	27
Rum2Bac	40	39
Rum2Bac	40	47
Rum2Bac	40	47
Rum2Bac	40	48
Rum2Bac	40	42
Rum2Bac	40	38
Rum2Bac	40	29
Rum2Bac	40	36
Rum2Bac	40	49
Pig2Bac	10	8
Pig2Bac	10	12
Pig2Bac	10	10
Pig2Bac	10	8
Pig2Bac	10	6
Pig2Bac	10	12
Pig2Bac	10	5
Pig2Bac	10	7
Pig2Bac	10	6
Pig2Bac	10	6
Pig2Bac	10	9
Pig2Bac	10	10
Pig2Bac	10	8
Pig2Bac	10	8
Pig2Bac	10	8
Pig2Bac	10	12
Pig2Bac	10	17
Pig2Bac	10	8
Pig2Bac	10	4
Pig2Bac	10	7
Pig2Bac	10	10
Pig2Bac	10	7

Feral swine as sources of fecal contamination in recreational waters
Anna M. McKee, Paul M. Bradley, David Shelley, Shea McCarthy, and Marirosa Molina
Table A.2. Sample MST marker concentrations (gene copies per milliliter of water sample), number of Stx2 positive qPCR replicates (out of two) used for statistical analysis, the number of qPCR replicates (out of four) with estimated values below the limit of quantification, and citizen science measured *Escherichia coli* concentrations. †Mean MST marker concentration included at least one qPCR replicate below the limit of quantification with a final concentration estimated from a normal distribution centered around the qPCR-based concentration estimate (see Methods for a description of the estimation process). *E. coli* MPN was calculated as the mean MPN across three dilutions from McCarthy (2020).

Sample ID	Site Name	Date	HF183 copies/mL	HF183 estimated qPCR reps	Pig2Bac copies/mL	Pig2Bac estimated qPCR reps	Rum2Bac copies/mL	Rum2Bac estimated qPCR reps	Stx2 Count	*E. coli* MPN
SA001	MYERS CR	12/18/2017	0	0	0	0	0	0	0	NA
SA002	CEDAR CR ABV MYERS CR	12/18/2017	0	0	0	0	0	0	0	NA
SA003	DRY BRANCH	12/18/2017	0	0	0	0	0	0	0	NA
SA004	CEDAR CR @ GAGE	12/18/2017	0	0	11†	4	0	0	0	NA
SA005	CEDAR CR @ KINGSNAKE	12/18/2017	0	0	1†	3	0	0	0	NA
SA006	CEDAR CR @ KINGSNAKE	3/12/2018	0	0	16	0	0	0	0	NA
SA007	MYERS CR	3/12/2018	0	0	0	0	0	0	2	NA
SA008	CEDAR CR ABV MYERS CR	3/12/2018	0	0	0	0	0	0	0	NA
SA009	CEDAR CR @ GAGE	3/12/2018	0	0	82	0	0	0	2	NA
SA010	DRY BRANCH	3/12/2018	0	0	0	0	0	0	0	NA
SA011	BATES OLD RIVER	4/30/2018	0	0	5†	4	0	0	0	NA
SA012	CONGAREE R @ SC601	4/30/2018	1†	3	3†	4	0	0	0	NA
SA013	TOMS CR	4/30/2018	0	0	0	0	0	0	0	NA
SA014	CEDAR CR @ KINGSNAKE	4/30/2018	0	0	0	0	0	0	0	NA
SA015	MUCK SWAMP	4/30/2018	0	0	0	0	0	0	0	NA
SA016	CEDAR CR @ GAGE	4/30/2018	0	0	0	0	0	0	0	NA
SA017	WISE LAKE	4/30/2018	0	0	0	0	0	0	0	NA
SA018	WESTON LAKE	4/30/2018	0	0	1†	2	0	0	0	NA
SA019	DRY BRANCH	4/30/2018	0	0	0	0	0	0	0	NA
SA020	MYERS CR	4/30/2018	0	0	0	0	0	0	0	NA
SA021	CEDAR CR ABV MYERS CR	4/30/2018	0	0	0	0	0	0	0	NA

SA022	CONGAREE R @ SC601	5/21/2018	11†	4	0	0	0	0	0	NA
SA023	BATES OLD RIVER	5/21/2018	0	0	0	0	0	0	0	NA
SA024	TOMS CR	5/21/2018	0	0	19†	2	0	0	0	NA
SA025	CEDAR CR @ KINGSNAKE	5/21/2018	0	0	7	0	0	0	2	NA
SA026	MUCK SWAMP	5/21/2018	0	0	0	0	0	0	0	NA
SA027	DRY BRANCH	5/21/2018	0	0	0	0	0	0	0	NA
SA028	CEDAR CR @ GAGE	5/21/2018	6†	2	0	0	0	0	0	NA
SA029	WISE LAKE	5/21/2018	0	0	0	0	0	0	0	NA
SA030	WESTON LAKE	5/21/2018	0	0	0	0	0	0	0	NA
SA031	MYERS CR	5/21/2018	0	0	0	0	0	0	0	NA
SA032	CEDAR CR ABV MYERS CR	5/21/2018	0	0	0	0	0	0	0	NA
SA033	COOKS GUT	5/21/2018	0	0	0	0	0	0	2	NA
SA034	CONGAREE R @ GAGE	5/21/2018	2†	2	0	0	0	0	0	NA
SA035	TOMS CR	7/9/2018	3†	2	17†	2	0	0	0	NA
SA036	CEDAR CR @ KINGSNAKE	7/9/2018	0	0	6†	4	0	0	2	NA
SA037	CEDAR CR @ GAGE	7/9/2018	0	0	7†	3	0	0	0	NA
SA038	DRY BRANCH	7/9/2018	0	0	0	0	0	0	0	NA
SA039	MYERS CR	7/9/2018	0	0	0	0	0	0	0	NA
SA040	CEDAR CR ABV MYERS CR	7/9/2018	0	0	0	0	0	0	0	NA
SA041	CEDAR CR @ KINGSNAKE	9/10/2018	0	0	5†	2	0	0	0	NA
SA042	CEDAR CR ABV MYERS CR	9/10/2018	1†	3	0	0	0	0	0	NA
SA043	MYERS CR	9/10/2018	2†	3	10†	1	0	0	0	NA
SA044	DRY BRANCH	9/10/2018	0	0	0	0	0	0	0	NA
SA045	CEDAR CR @ GAGE	9/10/2018	5†	4	18	0	0	0	1	NA
SA046	TOMS CR	9/10/2018	0†	2	101	0	0	0	2	NA
SA047	MYERS CR	10/22/2018	5†	4	2†	4	0	0	0	308
SA048	CEDAR CR ABV MYERS CR	10/22/2018	0	0	0	0	0	0	0	16
SA049	CEDAR CR @ KINGSNAKE	10/22/2018	0	0	127	0	0	0	2	161
SA050	CEDAR CR @ GAGE	10/22/2018	1†	3	391	0	0	0	0	194
SA051	DRY BRANCH	10/22/2018	0	0	0	0	0	0	0	2
SA052	CEDAR CR @ GAGE	2/11/2019	0	0	22	0	0	0	0	67
SA053	DRY BRANCH	2/11/2019	0†	2	1†	3	0	0	0	4
SA054	MYERS CR	2/11/2019	0	0	0	0	0	0	0	84
SA055	CEDAR CR ABV MYERS CR	2/11/2019	0	0	0	0	0	0	0	NA
SA056	CEDAR CR @ KINGSNAKE	2/11/2019	0	0	14	0	0	0	0	43

SA057	DRY BRANCH	3/7/2019	2†	3	3†	4	0	0	2	NA
SA058	CEDAR CR @ KINGSNAKE	3/7/2019	0†	2	106	0	0	0	2	530
SA059	MYERS CR	3/7/2019	0	0	0	0	0	0	2	427
SA060	CEDAR CR ABV MYERS CR	3/7/2019	0†	3	0	0	0	0	0	63
SA061	WATEREE R	4/1/2019	5†	4	2†	2	0	0	0	NA
SA062	HORSESHOE LAKE	4/1/2019	2†	3	4†	4	0	0	0	NA
SA063	CONGAREE R @ SC601	4/1/2019	12†	4	4†	1	0	0	2	NA
SA064	BATES OLD RIVER	4/1/2019	0	0	22†	3	0	0	0	NA
SA065	STUMP GUT	4/1/2019	7†	4	5†	1	0	0	0	54
SA066	CEDAR CR @ GAGE	4/1/2019	1†	3	398	0	5†	4	0	309
SA067	WISE LAKE	4/1/2019	0	0	6†	1	0	0	0	NA
SA068	WESTON LAKE	4/1/2019	0	0	10†	4	0	0	0	NA
SA069	CEDAR CR ABV CONGAREE R	4/1/2019	6†	4	5†	2	0	0	0	83
SA070	DRY BRANCH	4/1/2019	0	0	0	0	0	0	0	24
SA071	CONGAREE R ABV CEDAR CR	4/1/2019	10†	4	7†	2	0	0	0	59
SA072	CEDAR CR @ KINGSNAKE	4/1/2019	0	0	0	0	0	0	0	79
SA073	MYERS CR	4/1/2019	0	0	0	0	4†	4	2	174
SA074	CEDAR CR ABV MYERS CR	4/1/2019	0	0	0	0	0	0	0	6
SA075	CONGAREE R @ GAGE	4/1/2019	26	0	0	0	0	0	0	NA
SA076	COOKS GUT	4/1/2019	11†	4	0†	2	0	0	0	NA
SA077	CEDAR CR @ GAGE	6/11/2019	0	0	43	0	3†	4	2	591
SA078	DRY BRANCH	6/11/2019	0	0	0	0	0	0	0	33
SA079	MYERS CR	6/11/2019	0	0	3†	3	9†	4	0	348
SA080	CEDAR CR ABV MYERS CR	6/11/2019	0	0	0	0	0	0	0	99
SA081	CEDAR CR @ KINGSNAKE	6/11/2019	1†	2	4	0	0	0	2	332
